# Improved Multiple Displacement Amplification (iMDA) and Ultraclean Reagents

**DOI:** 10.1186/1471-2164-15-443

**Published:** 2014-06-06

**Authors:** S Timothy Motley, John M Picuri, Chris D Crowder, Jeremiah J Minich, Steven A Hofstadler, Mark W Eshoo

**Affiliations:** Ibis Biosciences an Abbott Company, 2251 Faraday Ave. Suite 150 Carlsbad, CA 92008 USA

**Keywords:** Whole genome amplification, Next generation sequencing, Multiple displacement amplification, Contamination, Clean reagents, DNA-free

## Abstract

**Background:**

Next-generation sequencing sample preparation requires nanogram to microgram quantities of DNA; however, many relevant samples are comprised of only a few cells. Genomic analysis of these samples requires a whole genome amplification method that is unbiased and free of exogenous DNA contamination. To address these challenges we have developed protocols for the production of DNA-free consumables including reagents and have improved upon multiple displacement amplification (iMDA).

**Results:**

A specialized ethylene oxide treatment was developed that renders free DNA and DNA present within Gram positive bacterial cells undetectable by qPCR. To reduce DNA contamination in amplification reagents, a combination of ion exchange chromatography, filtration, and lot testing protocols were developed. Our multiple displacement amplification protocol employs a second strand-displacing DNA polymerase, improved buffers, improved reaction conditions and DNA free reagents. The iMDA protocol, when used in combination with DNA-free laboratory consumables and reagents, significantly improved efficiency and accuracy of amplification and sequencing of specimens with moderate to low levels of DNA. The sensitivity and specificity of sequencing of amplified DNA prepared using iMDA was compared to that of DNA obtained with two commercial whole genome amplification kits using 10 fg (~1-2 bacterial cells worth) of bacterial genomic DNA as a template. Analysis showed >99% of the iMDA reads mapped to the template organism whereas only 0.02% of the reads from the commercial kits mapped to the template. To assess the ability of iMDA to achieve balanced genomic coverage, a non-stochastic amount of bacterial genomic DNA (1 pg) was amplified and sequenced, and data obtained were compared to sequencing data obtained directly from genomic DNA. The iMDA DNA and genomic DNA sequencing had comparable coverage 99.98% of the reference genome at ≥1X coverage and 99.9% at ≥5X coverage while maintaining both balance and representation of the genome.

**Conclusions:**

The iMDA protocol in combination with DNA-free laboratory consumables, significantly improved the ability to sequence specimens with low levels of DNA. iMDA has broad utility in metagenomics, diagnostics, ancient DNA analysis, pre-implantation embryo screening, single-cell genomics, whole genome sequencing of unculturable organisms, and forensic applications for both human and microbial targets.

## Background

Next-generation DNA sequencing (NGS) typically requires nanogram to microgram levels of DNA. Many specimens of interest have insufficient amounts of nucleic acids for direct sequencing. To sequence these samples one must amplify the DNA without altering the representation of the original DNA sample. A widely used method for whole genome amplification is multiple displacement amplification (MDA); MDA relies on priming of target DNA with random primers and the use of the strand-displacing φ29 polymerase to amplify all of the DNA in a given sample [1–3]. φ29 DNA polymerase is a highly processive, strand-displacing polymerase with a very low error rate of 1 in 10^6^-10^7^ nucleotides [4, 5]; the error rates of Taq polymerase and Pfu polymerase, both commonly used in PCR are 3 in 10^4^ and 3 in 10^6^, respectively [6, 7]. Recently a method for the whole genome amplification of DNA from single cells called MALBAC was reported to perform better than MDA [8]. This method employs several rounds of multiple primer annealing extension cycles with a strand-displacing polymerase followed by PCR. Another report describes the use of MDA in nanoliter-scale polydimethylsiloxane microwells produced in a microfabrication facility [9]. Despite total reaction volumes of 12 nl, the segregation of intact bacterial cells does not result in total fluidic isolation, requires nanoliter liquid handling capabilities and loading cells at a density that results in 90% empty wells. In this study, we report improvements to typical φ29-based MDA protocols through the addition of a second strand-displacing DNA polymerase, improved reaction formulation and conditions. Previous studies have shown that φ29-based amplification in the presence of a second strand-displacing DNA polymerase improves DNA microarray sensitivity relative to multiplex PCR amplification or amplification with φ29 DNA polymerase alone [10]. Our iMDA protocol does not require the use of FACS, specialized microfabrication or operating with nanoliter volumes yet provides an ultraclean DNA amplification reaction.

A number of MDA kits are commercially available; however, these kits generally recommend 10 ng of template DNA. This requirement stems from the fact that reagents included with the kits contain contaminating DNA that compete with the amplification of template of interest [11]. Sensitivity of MDA can be significantly improved by employing reagents that are free of contaminating DNA. In addition, laboratory consumables can also be a source of contaminating DNA that can confound genetic analyses. Between 1993 and 2009 the "phantom of Heilbronn" was one of Germany's most wanted criminals; this female DNA profile was found in samples collected at over 40 crime scenes [12]. After inconsistent results led to a more thorough investigation, the DNA profile was found to belong to a woman working in the factory that made swabs used to collect DNA evidence [12]. There have also been reports of DNA contamination in nucleic acid extraction columns with both mouse-specific nucleic acids and xenotropic murine leukemia virus-related virus (XMRV) were detected in the eluants from new naïve columns [13, 14]. These reports could be dismissed as isolated incidents except that upon screening of public, non-primate nucleic acid sequence databases such as NCBI, Ensembl, JGI, and UCSC, contamination with the primate-specific element AluY was found in 492 of 2749 database entries, suggesting widespread human DNA contamination in studies employing DNA sequencing [15].

In previous work by others, enzyme production methods have been modified to reduce contaminating DNA [11]. In addition, PCR amplification buffers and enzymes have been decontaminated by treatment with heat-sensitive DNA nucleases [16]. UV irradiation has also been used to decontaminate reagents and laboratory disposables used in MDA [17] but is of limited value as the extent of the DNA degradation by the UV light decreases with the square of the distance from the UV light source and can have a negative effect on the properties of the reagents and materials treated. Laboratory disposables have also been decontaminated with ethylene oxide (ETO) [18]. The effectiveness of both UV or ethylene oxide treatment has been limited for nucleic acid decontamination as the cell walls and membranes of an organism can serve to protect the cellular DNA [19, 20].

In this study we report the development and performance of methods for producing ultraclean iMDA reactions that are especially well suited for whole genome analyses by NGS. As part of these studies we developed an ethylene oxide protocol for the decontamination of laboratory consumables that inactivates free DNA as well as dried cellular DNA. The ultraclean reagents and consumables enabled the amplification of trace levels of target DNA while maintaining both genomic representation and balance of the starting DNA sample.

## Results

### Decontamination of laboratory consumables with ETO

To determine the effectiveness of the ETO treatment, laboratory consumables were contaminated with bacterial DNA, whole bacterial cells, or human DNA. *K. pneumoniae* DNA was dried onto pipette tips prior to the ETO treatment protocol. Four identical sets of ten contaminated pipette tips were packaged into individual test boxes. Three of these test boxes were subject to the ETO protocol. The contaminating DNA was recovered by rinsing the pipette tips repeatedly with warm Tris-EDTA buffer. The quantity of the DNA recovered from the pipette tips was determined with a *K. pneumonia*-specific qPCR assay. From the tips not treated with ETO, the average amount of DNA recovered was 21 ng per tip. Of the fifteen tips treated with ETO, all had *K. pneumonia* DNA below the limit of detection of the qPCR assay (0.350 pg) representing a greater than 6x10^4^ fold reduction in the level of detectable DNA (Table 1).

**Table 1:** **Decontamination of laboratory consumables with ethylene oxide treatment**

		DNA recovered	
	Contamination type	No treatment (n)	ETO treated (n)
Pipette tips	Bacterial DNA	21 ng ± 3 (5)	BLD* (15)
Microcentrifuge	20 ul Bacterial cells		
Tubes		90 ng ± 11 (4)	BLD* (15)
Microcentrifuge	100 ul Bacterial cells		
Tubes		402 ng ± 112 (3)	BLD* (2)
Extraction columns	Free DNA-human	10 ng ± 2 (3)	BLD* (3)

*BLD = below the limit of detection (LOD).

To determine the effectiveness of the ETO treatment protocol for the elimination of cellular DNA, *B. cereus* overnight cultures in rich media (20-μl and 100-μl aliquots) were dried in micro-centrifuge tubes and subject to ETO treatment in differing locations in the treatment chamber. The nucleic acids were recovered from the tubes, and the level of *B. cereus* DNA was determined using a *Bacillus*-specific qPCR assay. Between 79 ng and 101 ng of *Bacillus* DNA was recovered from non-ETO-treated tubes contaminated with the 20-μl samples and between 290 ng and 514 ng was recovered from tubes contaminated the 100-μl aliquots (Table 1). All ETO-treated tubes that had been contaminated with *Bacillus* cells had DNA below the limits of detection of the qPCR assay (0.72 pg) representing a >10^5^ fold reduction in the amount of detectable DNA following ETO treatment. Based upon the performance of the exogenous qPCR internal positive control there was no indication of qPCR inhibition.

Macherey-Nagel nucleic acid extraction columns were contaminated with 20 ng of human genomic DNA and treated with the ETO protocol. DNA was recovered from the columns, and the level of human DNA was determined with human Alu-specific qPCR. There was a >12,000 fold reduction in the DNA recovered from the ETO-treated vs. untreated contaminated columns (Table 1). The ETO-treated columns were also tested to assure that the ETO treatment did not affect performance. Three treated and three untreated columns were used to extract 100 ng of *K. pneumoniae* DNA with no significant difference in performance (85.4% ± 3.9% recovery with ETO-treated columns vs. 90.2% ± 0.8% recovery from untreated columns).

### Comparison of sensitivities of iMDA and commercial MDA kits

**Figure 1:**
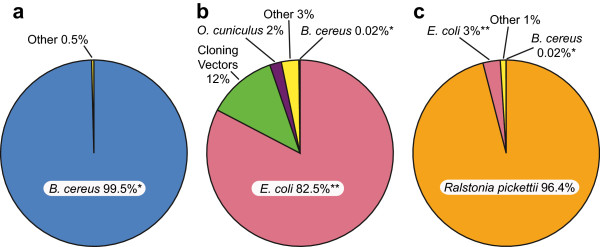
**Metagenomic sequence analysis of 10 fg of** ***B. cereus*** **genomic DNA amplified by (a) the Ibis Ultraclean iMDA protocol or with commercial WGA Kits (b) Genomiphi V2 WGA or (c) Qiagen REPLI-g WGA.** All amplified reactions were sequenced by ion semiconductor sequencing (Ion Torrent PGM) followed by metagenomic analysis. *All reads that mapped to the *B. cereus* clade (i.e., *B. cereus, B. thuringiensis*, and *B. anthracis*) are reported as *B. cereus*. **All reads that mapped to *Escherichia* or *Shigella* are reported as *E. coli*.

The sensitivity and specificity of the iMDA protocol was compared to sensitivity and specificity of two different commercial MDA kits: Qiagen REPLI-g® UltraFast Mini Kit and GenomiPhi V2 DNA Amplification Kit. For these tests, a very low level of *B. cereus* bacterial genomic DNA (10 fg, equivalent to the expected DNA from ~1-2 bacterial cells) was used as a template in the amplification reactions. The resulting amplified DNA was sequenced on an Ion Torrent semiconductor sequencing system. The DNA sequences from each reaction were subject to analysis with the Ibis Galaxy Analysis software to determine their meta-genomic species composition; the identified species in each sample were used to construct the pie-charts shown in Figure 1. The iMDA sequencing reactions produced 1.14 × 10^6^ reads (average read length of 118 bases) with >1.13 × 10^6^ of the reads specifically identified by the Ibis Galaxy analysis. The iMDA template genome, *B. cereus* was identified as the source of >99.4% (*B. cereus* specific reads/total mapped reads) of the mapped reads with 0.2% of the reads mapping to other *Bacillus* clade species.

Sequencing of the Qiagen REPLI-G MDA amplified DNA produced 4.1 x 10^5^ total reads (average read length of 114 bases) with 99.8% of the reads being mapped by the Ibis Galaxy analysis. The vast majority (>96.3%) of the reads mapped to *Ralstonia pickettii*, 3% mapped to *E. coli* or *Shigella*, and only 0.02% mapped to the template genome*, B. cereus.* Due to the close genetic similarity of *E. coli* and *Shigella* species, these reads were combined in Figure 1. Sequencing of the Genomiphi V2 MDA amplified DNA generated 1.36 x 10^6^ total reads (average read length of 114 bases) with 1.35 x 10^6^ reads being mapped by the Ibis Galaxy analysis. The majority of the reads mapped to *E. coli* (76.4%) with an additional 5.8% mapping to *Shigella* species. Again, because of the close genetic similarity of *E. coli* and *Shigella* species, these reads were combined in Figure 1. Only 0.02% mapped to the actual template*, B. cereus*. The identification of *O. cuniculus* in the Genomiphi V2 reactions was confirmed by collecting those specific reads (27,896) and using a separate metagenomic BLAST analysis. Further analysis showed that of the reads matching *O. cuniculus*, 80% mapped to the *alpha-globin* gene.

The level of relative sensitivity of the iMDA reaction was compared to the REPLI-G WGA and Genomiphi V2 WGA by dividing the percentage of reads mapped to *B. cereus* in iMDA (99.5%) by the percentage of reads mapped to *B. cereus* in the commercial WGA reactions (0.02%). From this analysis, the iMDA reaction was nearly 5000 fold more sensitive than the REPLI-g WGA and the Genomiphi V2 WGA when there was a low level of input template.

### Genomic representation analysis of iMDA DNA by NGS

**Figure 2:**
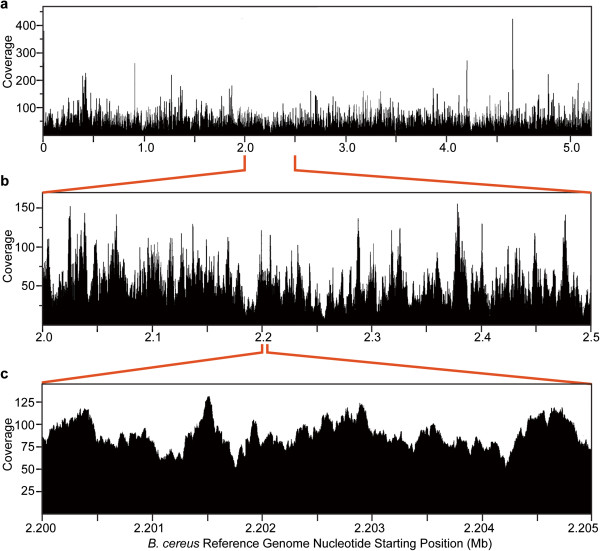
**Whole genome coverage of iMDA DNA.** One picogram (pg) of *B. cereus* genomic DNA was amplified by the iMDA protocol and the amplified DNA was sequenced. Plots show depth of coverage vs. the position in the reference genome at increasing magnifications. **(a)** Mapping across the entire reference genome. **(b)** Mapping from 2 MB to 2.5 MB in the reference genome. **(c)** Mapping from 2.200 MB to 2.205 MB in the reference genome.

**Figure 3:**
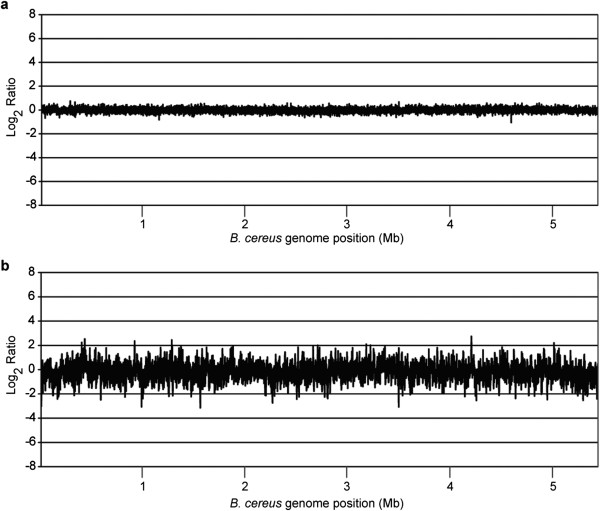
**Relative sequencing balance across the** ***B. cereus*** **genome. (a)** Comparison of balance of two independent libraries made from 1 μg of *B. cereus* genomic DNA in 500-bp bins without iMDA. **(b)** Comparison of balance between iMDA DNA obtained from 1 pg of *B. cereus* genomic DNA template vs. 1 μg of genomic DNA across the genome in 500-bp bins.

To assess whether the iMDA method achieves relatively complete coverage of the input genome, a non-stochastic amount of *B. cereus* template DNA (1 pg) was amplified (11,638,000 fold) in a 2-h iMDA reaction. An aliquot of the iMDA reaction and two independent non-amplified *B. cereus* genomic DNA (1 μg) samples were used to produce sequencing libraries. The sequencing reactions were mapped to the *B. cereus* ATCC 10987 (NC_003909.8) published sequence with NextGENe software from SoftGenetics. The results are summarized in Table 2. Sequence data obtained from the iMDA template and from both of the genomic templates covered more than 99.98% of the reference genome at least 1X coverage) and greater than 99.9% of the genome with at least 5X coverage. The average read lengths were comparable (212 to 224 bases) as were the total number of bases read (398 MB for the iMDA template vs. 574 MB for the genomic DNA). The average coverage of the iMDA template was 64 fold, whereas that of the purified genome was 105 fold. Figure 2 depicts the coverage vs. position for the iMDA data at various degrees of resolution; these data demonstrate the uniformity of the genomic coverage indicating that the iMDA protocol did not introduce bias. The depth of coverage between iMDA and genomic DNA samples were compared in 500-bp bins across the genome (Figure 3). The average variation between the two samples of genomic DNA was 1.1 fold (±1.1). The average variation between iMDA and the genomic DNA was 1.5 fold (±1.4); for 82.7% of the sequence bins the variation was within 2 fold and for 99.0% of the bins variation was within 4 fold. This is especially notable as the iMDA sample was amplified more than 1.1x10^7^ fold. We were not able to make a comparable comparison for representation and coverage using commercial MDA kits. For example MDA utilizing the illustra genomiphi V2 kit with 1 pg of *B cereus* template DNA yielded only 37 ng of *B. cereus* DNA, as determined by qPCR. The total DNA yield of 10.9 μg of DNA indicated only 3.3x10^4^ fold amplification of the *B. cereus* DNA and less than 1% of the total amplified DNA was derived from the template DNA.

**Table 2:** **Whole genome sequence analysis of** ***B. cereus*** **genomic and iMDA DNA**

	Genomic-1	Genomic-2	iMDA-1 pg
**Total MB**	445	574	398
≥ **1X coverage**	99.978%	99.979%	99.975%
≥ **5X coverage**	99.941%	99.939%	99.898%
**Average read length**	224	213	212
**Average coverage**	82	105	64
**Total reads**	1989230	2696410	1879724
Mapped reads*	1988492	2695230	1879079
B. cereus**	99.80%	99.62%	99.64%

*Mapped reads were identified with the Ibis Galaxy analysis.

**Includes all reads that mapped to the *B. cereus* clade, (i.e., *B. cereus*, *B. thuringiensis*, and *B. anthracis*).

### Coverage uniformity across the genome

**Figure 4:**
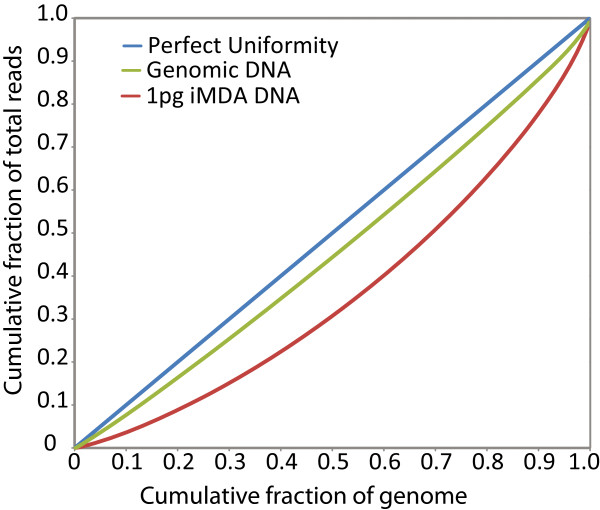
**Lorenz curve iMDA and genomic sequencing of** ***B.cereus*** . Lorentz curves depict the relative bias in average read coverage across the *B. cereus* genome. Each curve was calculated by dividing the genome into 500 bp bins, counting the average read depth across each bin, and using the resultant cumulative distribution function for read depth to determine the cumulative proportion of total genome coverage (y-axis) accounted for by the cumulative proportion of bins (x-axis). The ideal Lorentz curve (black line) for a distribution in which all of the bins have the same coverage is plotted for comparison.

In order to assess coverage uniformity and relative bias generated by the iMDA process we generated Lorenz curves from sequence derived from 1 pg of *B.cereus* genomic template amplified by iMDA and unamplified template. The results are shown in Figure 4 in which we compare the Lorenz curves from both samples at an average coverage depth of 73X. The diagonal line indicates perfect uniformity of coverage and deviation indicates an uneven distribution of reads. It is evident that the iMDA provides a very high uniformity of genomic coverage and this is in good agreement with the 99.9% coverage value calculated in Table 2.

## Discussion

Advancements in NGS technologies are revolutionizing biology. The ability to generate very deep sequence analysis of a DNA sample in a very short time allows the investigation of many complex samples with meta-genomic analysis. However, any contaminants contained in the sample buffers, enzymes, or laboratory consumables will confound the analysis. There are many potential sources of contamination including pipette tips, tubes, extraction columns, and commercial enzymes and buffers. Contamination in any of these may negate even the most stringent contamination controls. We demonstrate here the ability to completely remove detectable levels of contaminating DNA on pipette tips, centrifuge tubes, and extraction columns with an optimized ethylene oxide treatment process. This process did not interfere with downstream applications.

Despite improvements in NGS sensitivity, there is still the requirement for a significant amount of template DNA; typically 100 ng to 5000 ng of input DNA. Many important specimens contain DNA at levels far below this threshold. To address this problem, a number of methods have been developed to amplify DNA for NGS. However, these technologies are limited by the level of contaminating DNA in enzymes, buffers, and reagents used during the amplification and by their abilities to maintain representation of the starting sample. In some cases individual cells have been successfully isolated, amplified, and sequenced, but this typically required multiple rounds of fluorescence-activated cell sorting (FACS) [21], micromanipulation [22], or micro-fluidics [23] to separate the intact, individual cells. Although these are powerful techniques, many samples of interest contain free DNA and cannot be isolated in this manner. Also, the amplification technology can introduce tremendous bias in the sequencing coverage [17]. Most NGS technologies also employ a clonal amplification step, which can result in further bias. Commercially available MDA kits can be used to amplify DNA for NGS but typically recommend >10 ng of initial template. Even the recent single cell genomics studies start with ~5 pg, the genomic DNA equivalent of a single eukaryotic cell [24]. This study compared the combination of ultraclean reagents and the iMDA protocol with two commercial MDA kits. In this study we have developed methods for preparation of ultraclean reagents and have developed an improved multiple displacement amplification protocol that enables the successful amplification of 10 fg of DNA template – 1,000,000 fold less than required by commercial kits.

The iMDA protocol has several features that, in combination, improve its performance over a standard MDA reaction. First the iMDA reaction employs φ29 DNA polymerase and a second strand-displacing polymerase Klenow exo-. Though Klenow exo- is not a proof reading polymerase it has an error rate that is comparable to Taq [25]. The combined use of φ29 and Klenow has been previously shown to improve amplification compared to amplification by φ29 alone with DNA microarrays [10]. DNA amplification by Klenow exo- used in strand displacement amplification (SDA) is not limited to higher molecular weight templates like MDA using φ29 DNA polymerase [3, 26]. We posit that the use of these two strand-displacing DNA polymerases may complement each other to better amplify templates of varying lengths. The iMDA reaction buffer also contains high levels of the thermo-protectant trehalose, which enables the reaction to be performed at 37°C vs. 30°C. Trehalose has also been previously shown to improve MDA performance [27]. The iMDA reaction buffer also contains high levels of non-ionic detergent which improved the yields of the reactions [11]. Lastly the iMDA protocol also employs, random septamers rather than hexamers to provide a correspondingly higher Tm and longer primer length has been shown to reduce the Km of Klenow fragment potentially improving its performance in iMDA [28]. The iMDA protocol employs a very high fidelity proof reading DNA polymers, φ29, and a non-proof reading polymerase with an error comparable to Taq DNA polymerase Use of DNA amplified using the iMDA protocol in NGS reactions resulted in greater coverage across the genomic template than did commercially available MDA kits and maintained representative balance especially when employing processes for removing background DNA in the reagents. Combined with ethylene oxide treatment protocol for the decontamination of the laboratory consumables, developed in this study, the sequencing coverage and sequencing accuracy from specimens with low levels of DNA is greatly improved.

By employing this combination of strategies, the iMDA method outperforms published MDA in the specific amplification of target template and uniformity of coverage indicating very little introduction of bias even with extreme amplification [11]. The iMDA protocol does not require extensive enzyme purification or microfabrication facilities and enables the use of standard reaction volumes. This combination of ultraclean consumables and reagents, along with improvements incorporated into iMDA provides the opportunity for specific detection and genomic sequence analysis from samples that were previous not possible due to template limitation.

## Conclusions

The iMDA reaction developed in this study significantly outperformed commercial MDA kits in the ability to amplify specimens with low levels of DNA. and importantly the iMDA protocol maintains balance and representation. The iMDA protocol combined with ultra-clean reagents and consumables has broad utility for metagenomics, molecular diagnostics, ancient DNA analysis, pre-implantation embryo screening, whole genome analysis of circulating tumor cells, and forensic applications for both human and microbial targets. Use of this protocol will make in-depth genetic analysis of extremely low level DNA templates, including those derived from single cells and viruses, possible.

## Methods

### Bacterial culture, nucleic acid preparation, and qPCR

*B. cereus* (ATCC 10987) was aerobically cultured in LB liquid medium (Becton Dickenson, Franklin Lakes, NJ) at 30°C for 18 h. The number of colony forming units (CFU) per μl, determined by plating, was ~3,000. Aliquots of this culture (20 μl or 100 μl) were dried in sterile, 2-ml screw cap microcentrifuge tubes (Sarstedt, Germany). *B. cereus* DNA was recovered from the tubes by adding 250 μl of 10 mM Tris, pH 7.5, 0.05 mM EDTA that had been incubated at 50°C, vortexing for 30 seconds, and incubating at 95°C for 15 min, followed by another 60 seconds of vortexing. The solution was chilled on ice and diluted 1:100 with 10 mM Tris, pH 7.5, 0.05 mM EDTA and used directly in qPCR.

Bacterial DNA for contamination studies of the pipette tips was prepared from *K. pneumonia* (ATCC 13883). Cells were grown overnight on nutrient agar plates (Becton Dickenson). DNA was extracted using the Wizard Genomic DNA Purification Kit (Promega, Madison, WI) according to the manufacturer’s protocol. This DNA (3 μg) was used in a 40-ml iMDA reaction with a 6-h incubation at 37°C to yield ~40 mg of amplified DNA. The resulting amplified DNA was diluted to 10 ng/μl and used to contaminate 200-μl pipette tips (Mettler-Toledo, Columbus, OH) by pipetting 100 μl of the DNA solution into each tip ten times with a one second hold during the fill step of each cycle. The solution was ejected, and the tips were allowed to dry in a biosafety cabinet. To obtain test samples, each tip was washed ten times with 120 μl of 50°C 10 mM Tris, pH 7.5, 0.05 mM EDTA.

Human DNA (Promega) was used to contaminate Nucleospin Blood Columns (Macherey-Nagel, Bethlehem, PA). The DNA was diluted to a concentration of 1 ng/μl in 10 mM Tris, pH 7.5, 0.05 mM EDTA and 20 μl was spotted onto the center of each of the extraction membranes. The DNA was allowed to dry on the columns overnight. To obtain test samples of DNA from the columns, 75 μl of 55°C 10 mM Tris, pH 7.5, 0.05 mM EDTA was added to the columns followed by a 15-min incubation at room temperature. Columns were centrifuged for three minutes at 6,000 × g. The eluate was removed to a separate tube, and the column wash step was repeated with a 3-min, 14,000 × g recovery spin. The eluants were combined.

All DNA stocks were quantified by organism-specific qPCR. All qPCR reactions were performed and analyzed on a StepOnePlus™ Real-Time PCR system (Life Technologies, Carlsbad, CA). qPCR cycling conditions for all qPCR assays were as follows: 95°C for 10 min, 45 cycles of denaturation 95°C for 15 s, and annealing/extension 60°C for 1 min in a 25-μl reaction containing Brilliant II QPCR Master Mix with High ROX (Agilent Technologies, Wood Dale, IL), 500 nM of each primer, and 200 nM probe. All reactions were performed in duplicate with four negative (no template) controls (NTC) and 16 standards prepared from dilutions of extracted OD_260_ quantified genomic DNA. An internal positive control (Life Technologies) was included to allow detection of any inhibition by the material recovered from the ETO-treated tubes and pipette tips. *K. pneumonia* DNA was quantified using the following primers and probes: forward, AGCGCAACCCTTATCCTTTGT; reverse, CACTGGCAGTCTCCTTTGAGTTC; probe, FAM-CCAGCGGTTAGGCC-MGB. *B. cereus* DNA was quantified using the following primers and probes: forward, TGAAGGAGACATGGGTGACTCA; reverse, TGATTGCACCTGAAAGTTTACGA; probe, FAM-CGTAGGTTTACAAGCTCGTCTAATGTCTCAAGCAC-TAMRA. A previously described human Alu Yd6 qPCR protocol was used to quantitate human DNA [29]. The limits of detection (LODs) for the DNA on tips and tubes was0.35 pg, for *K. pneumoniae* DNA, 0.72 pg for cellular *B. cereus* DNA and 1.0 pg for human DNA.. These values include the extract dilution used in the qPCR assay and the analytical LODs for all qPCR assays was 10–20 fg.

### Improved multiple displacement amplifications (iMDA)

All iMDA reactions were performed in 100-μl volume and contained 50 mM Tris, pH 7.6 (Sigma-Aldrich Corp., St. Louis, MO), 12 mM MgCl_2_ (Sigma-Aldrich), 10 mM (NH_4_)_2_SO_4_ (Sigma-Aldrich), 0.57 M trehalose dihydrate (HPLC grade, Sigma-Aldrich), 1.1% V/V Tween 40 (Sigma-Aldrich), 2.8 mM dNTP mix (Bioline, Taunton, MA), 4 mM dithiothreitol (Life Technologies), 50 μM random septamer with a single 3’ phosphorothioate linkage (IDT, Coralville, IA), and 2 ng/μl sonicated polyadenylic acid (Abbott Molecular, DesPlaines, IL). After the addition of template DNA, the reactions were incubated at 95°C for 1 min followed by a cooling to 4°C in an MJ Thermocycler (Bio-Rad Laboratories, Hercules, CA). One hundred units of φ29 DNA polymerase (100U/μl, custom formulation of available stock, New England Biolabs, Ipswich, MA) and 50 units (50U/ul) of Klenow fragment exo- (New England Biolabs) were added, and the reactions were incubated at 37°C for 2 h followed by a 10-min incubation at 85°C. Clean production lots of amplification enzymes were screened and identified by using 10 fg bacterial DNA as template and showing >90% of the sequencing output using the amplified DNA was template derived. Ultra-clean iMDA reaction buffer without DTT, dNTPs, and primers was prepared by passing the buffer over a Q100 anion exchange membrane (Sartorius, Bohemia, NY) and through a 0.2-μm filter (Sartobran P 150, Sartorius) at a flow rate of 30 mL/min with the buffer collected and stored in a 500-mL IV bag (Metrix, Dubuque, IA) to maintain purity. Random septamers were dialyzed twice for 4 hours at 4°C against 4 L of solution containing 10 mM Tris, pH 8.0, and 50 μM EDTA using a 5-mL Float-A-Lyzer G2 with a 0.5–1 kDa MW cutoff (Spectrum Laboratories, Rancho Dominguez, CA); septamers were then passed through a Ultracel 30 K spin filter (Millipore Corp., Billerica, MA) and added to the iMDA buffer. Ultraclean nucleotides and 1 M DTT stocks were prepared by passing the solutions through a Ultracel 30 K spin filter (Millipore) before adding to the iMDA reaction mix. Individual lots of iMDA enzymes were screened for contaminating bacterial DNA by broad-range PCR and ESI-MS [30]. The LOD of this amplification when combined with NGS was less than 1 fg.

Commercial MDA assays, Qiagen REPLI-G Ultrafast WGA Reactions (Cat. no. 150033, Lot 142330089; Qiagen, Valencia, CA) and illustra GenomiPhi V2 DNA Amplification Kit (Cat. no. 25-6600-30, Lot 4683797; GE Healthcare Biosciences, Piscataway, NJ) were carried out in a 20-μl reaction volume according to manufacturers’ protocols with a 3-h incubation time at 30°C.

### Ethylene oxide treatment and materials

The consumables contaminated with bacterial or human DNA were sealed into ETO treatment pouches (Steris, Mentor, OH). The shrink wrap on new pipette tip boxes was slit ~2" with a clean single-edge razor blade. The 200-μl PCR tubes and 2.0-ml microcentrifuge centrifuge tubes were packed in plastic bottles with loose lids held in place with lab tape. All materials were packed into Steris-Isomedix Vis-U-All Self-Seal Pouches (STERIS). The bags were then packaged into cardboard boxes and shipped to Steris Isomedix for treatment. The ethylene oxide decontamination protocol was comprised of a preconditioning dwell time of >24 h at 115 °F, exposure to ethylene oxide at 14.7 inHgA at 125 °F for 5 h, followed by four successive nitrogen washes at 28 inHgA.

### Library preparation and ion semiconductor sequencing

DNA (1 μg) was fragmented by sonication using a Covaris S2 (Covaris, Woburn, MA), and libraries were prepared according to the Ion Plus Library Fragment Kit Protocol (Life Technologies) for either 100 or 200 base sequencing with 5 cycles of pre-OneTouch PCR enrichment during which the reaction was split into three tubes to minimize amplification bias. Size selection was performed with the 2.0% agarose Pippen Prep cassettes (Sage Scientific, Beverly, MA). Final library quality and quantity were assessed with the Agilent 2100 Bioanalyzer (Agilent Technologies) followed by Ion qPCR (Life Technologies). Libraries were amplified and enriched using the Ion OneTouch™ 100 or 200 base template kit (Life Technologies) according to the manufacturer’s standard protocol. Enriched Ion Sphere™ particles were sequenced on the Ion Torrent Personal Genome Machine (Life Technologies) using the Ion PGM™ Sequencing 100 or 200 Kit (Life Technologies) and 314 or 316 chips (Life Technologies). Base calling and subsequent FASTQ output was generated by the Ion Torrent Server v3.2.1 software (Life Technologies).

### Metagenomics and data analysis

The Ibis metagenomic analysis pipeline was developed from a customized workflow based on tools in the Galaxy Project ( http://galaxyproject.org) developed by Penn State and Emory University [31]. Prior to sequence analysis, reads of less than 100 bases were filtered from the data sets. The Ibis metagenomic analysis utilizes megablast (NCBI) results of each sequenced read and a weighting system to determine which organisms may have been present in a sample. The analysis runs megablast against a local NCBI GenBank database for each read with the following parameters: a word size of 16 and a maximum e-value of 1e-10. The e-value and GI accession number are recorded for each read matching an entry in GenBank. The GenBank tax-id for each read was then determined based upon the GI number and the species determined.

To determine the relative ‘uniqueness’ of each read a weighting system was used to decrease the contribution of sequences shared across multiple species compared to sequences specific to a given species. Following the weighting, all the votes were tallied across all the species and reads. The resulting tallies and percent of the total votes for each species are reported. Due do their high levels of homology reads that mapped to *B. cereus, B. anthracis*, and *B. thuringiensis* were reported as *B. cereus*[32]. To calculate the sequencing balance, the genome was divided into 500-bp bins, and the average fold coverage across the genome was determined for each data set (NextGENe, SoftGenetics, Inc., State College, PA). The fold coverage for each 500-bp bin was determined, and the fold coverage in each 500-bp bin was divided by the average fold coverage across the genome. The log 2 ratio of the genomic to genomic and the iMDA to genomic comparisons were calculated for each 500-bp bin and the ratios were then plotted across the genome.

For depicting the relative bias in average read coverage across the *B. cereus* genome we used a Lorez curve by dividing the genome into 500 bp bins, counting the average read depth across each bin, and using the resultant cumulative distribution function for read depth to determine the cumulative proportion of total genome coverage (y-axis) accounted for by the cumulative proportion of bins (x-axis). The ideal Lorenz curve (black line) for a distribution in which all of the bins have the same coverage is plotted for comparison.

## Availability of supporting data

The sequencing data supporting the results of this article are available in the NCBI sequencing repository under project SRP040249. ( http://www.ncbi.nlm.nih.gov/bioproject/241431).
